# Successful Rapid Desensitization to Ceftazidime/Avibactam in a Patient With Cystic Fibrosis and Multidrug‐Resistant *Pseudomonas aeruginosa* Pneumonia: A Case Report

**DOI:** 10.1002/ccr3.73208

**Published:** 2026-08-04

**Authors:** Zuoren Zhou, Jinqi Zhu, Ge Wu, Xi Kang, Qing Wang, Jie Liu, Yan Jiang

**Affiliations:** ^1^ Department of Respiratory and Critical Care Medicine The Affiliated Changsha Central Hospital, Hengyang Medical School, University of South China Changsha Hunan China; ^2^ Department of Pharmacy The Affiliated Changsha Central Hospital, Hengyang Medical School, University of South China Changsha Hunan China

**Keywords:** ceftazidime/avibactam, cystic fibrosis, drug hypersensitivity, multidrug‐resistant *Pseudomonas aeruginosa*, rapid desensitization

## Abstract

Ceftazidime/avibactam (CAZ‐AVI) is an important therapeutic option for multidrug‐resistant (MDR) *Pseudomonas aeruginosa* infections; however, hypersensitivity reactions may preclude its use and create significant therapeutic challenges. We report a 21‐year‐old woman with cystic fibrosis and recurrent MDR *P. aeruginosa* pneumonia who had a clinically suspected immediate hypersensitivity reaction to CAZ‐AVI. In the absence of effective alternative antimicrobial options, a 13‐step rapid intravenous desensitization protocol was performed under intensive monitoring. The patient subsequently tolerated full‐dose CAZ‐AVI therapy with marked clinical improvement. Rapid desensitization may represent an effective rescue strategy for patients with CAZ‐AVI allergy when applied under strict clinical indications and multidisciplinary supervision.

## Key Clinical Message

Rapid desensitization can enable safe administration of ceftazidime/avibactam therapy in critically ill patients with multidrug‐resistant Gram‐negative infections and clinically suspected immediate hypersensitivity when no equally effective alternatives are available.

## 1 Introduction

Cystic fibrosis (CF) is an autosomal recessive genetic disorder caused by mutations in the CFTR gene, leading to progressive structural lung disease, pancreatic insufficiency, and multisystem complications [1]. Abnormal mucus production and impaired mucociliary clearance predispose patients to chronic airway infections [2]. Among the causative pathogens, *P. aeruginosa* is one of the most prevalent and difficult to eradicate, owing to its ability to form biofilms, produce multiple virulence factors, and exhibit intrinsic antibiotic resistance [3]. As the disease progresses, MDR strains—particularly carbapenem‐resistant *P. aeruginosa* —frequently emerge, further limiting therapeutic options and contributing to poor clinical outcomes and increased mortality [4, 5].

Ceftazidime/avibactam (CAZ‐AVI) is a combination of a third‐generation cephalosporin and a novel β‐lactamase inhibitor. Avibactam inhibits Ambler class A, class C, and selected class D β‐lactamases, thereby restoring the activity of ceftazidime against selected multidrug‐resistant Gram‐negative pathogens [6, 7]. In *P. aeruginosa* , the activity of CAZ‐AVI is mainly relevant to resistance mechanisms such as AmpC β‐lactamase activity, extended‐spectrum β‐lactamases, and altered permeability or efflux, although susceptibility remains isolate‐dependent. CAZ‐AVI has been approved by the US Food and Drug Administration (FDA) and the European Medicines Agency (EMA) for the treatment of hospital‐acquired pneumonia and other serious infections. It has become an important therapeutic option for selected cases of MDR *P. aeruginosa* pneumonia.

However, when a patient develops suspected hypersensitivity to this agent, clinical management becomes particularly challenging. Rapid drug desensitization (RDD) may enable patients with immediate hypersensitivity reactions to safely receive first‐line therapy, thereby avoiding the need to switch to alternative agents that may be less effective, more costly, or associated with greater toxicity [8].

## 2 Case History and Examination

A 21‐year‐old woman with a 7‐year history of CF was admitted for worsening respiratory symptoms. She was born to consanguineous parents, and genetic testing confirmed a homozygous CFTR mutation (NM‐000492.4: c.1000C>T, p.R334W) [9]. Since the age of 14, she had experienced recurrent episodes of copious purulent sputum, fever, intermittent hemoptysis, and exertional dyspnea. She had been repeatedly hospitalized for bronchiectasis‐associated infection and *Pseudomonas aeruginosa* pneumonia. Recent antimicrobial susceptibility testing demonstrated susceptibility only to CAZ‐AVI and polymyxins, consistent with a multidrug‐resistant strain. However, she had previously developed immediate hypersensitivity reactions to CAZ‐AVI and cefoperazone/sulbactam, characterized by localized urticaria and flushing within 3 min of infusion that rapidly progressed to generalized involvement. Polymyxin B administration had caused immediate throat numbness and dyspnea. These findings were considered most consistent with immediate hypersensitivity reactions to multiple antimicrobial agents based on clinical history and temporal association with drug administration; however, formal allergological confirmation was not available.

She was readmitted on January 25, 2026, presenting with fatigue, conjunctival edema, tachypnea (28 breaths/min), tachycardia (127 bpm), and hypoxemia (SpO_2_ 90% on 4 L/min oxygen via nasal cannula at rest). Lung auscultation revealed coarse breath sounds and diffuse crackles. Digital clubbing was present without peripheral edema. Arterial blood gas analysis demonstrated type II respiratory failure with respiratory acidosis.

## 3 Methods (Differential Diagnosis, Investigations and Treatment)

**FIGURE 1:**
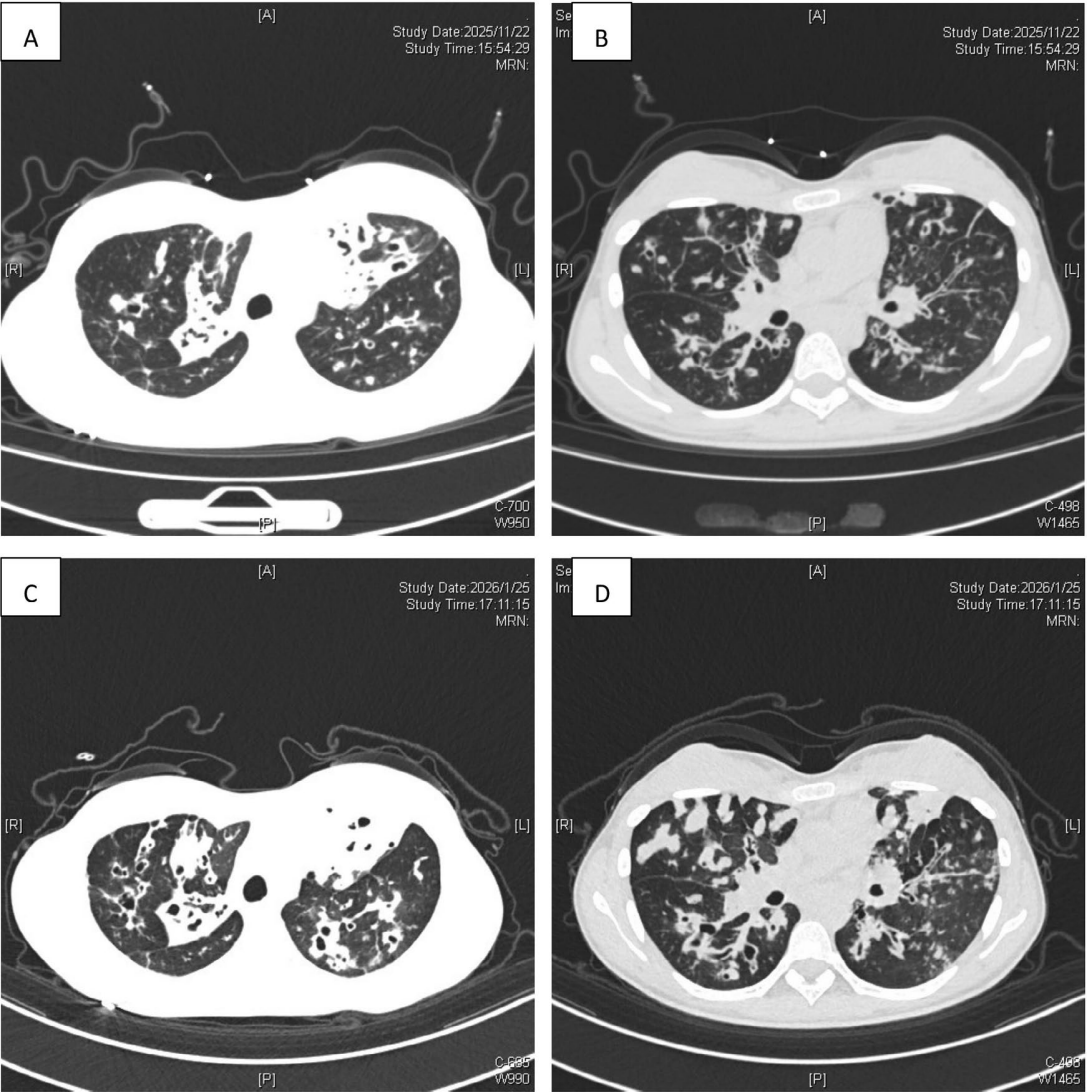
HRCT comparison between the previous (A, B) and current (C, D) hospitalizations. Panels (A, C) and (B, D) represent matched anatomical levels, respectively. The current images show marked progression of bilateral bronchiectasis, mucus plugging, and pulmonary consolidation, consistent with aggravated pulmonary infection.

Computed tomography pulmonary angiography (CTPA) demonstrated normal opacification of the main pulmonary artery, bilateral pulmonary arteries, and their branches, without filling defects, stenosis, dilatation, or vascular malformations, thereby excluding pulmonary embolism. Dedicated HRCT (Figure 1) demonstrated diffuse cystic and cylindrical bronchiectasis, interstitial fibrosis, newly developed multifocal peribronchial ground‐glass opacities, confluent consolidations, increased mucus plugging, and focal atelectatic changes, consistent with aggravated pulmonary infection. Sputum cultures were positive for *P. aeruginosa* . Empirical therapy with meropenem and amikacin was ineffective. Several alternative therapies were considered before selecting CAZ‐AVI desensitization. Cefiderocol was considered but was not selected because it is a cephalosporin‐class antibiotic and therefore carried a potential risk of recurrent immediate hypersensitivity in this patient with suspected cephalosporin‐related reactions. In addition, cefiderocol was not reimbursed, was more costly than CAZ‐AVI locally, and was unavailable in our hospital formulary, limiting timely access during severe respiratory failure. Imipenem/cilastatin/relebactam and meropenem/vaborbactam were similarly limited by high cost and poor local availability. Tobramycin inhalation had previously been administered repeatedly for 1–3‐month courses but failed to prevent recurrent pulmonary exacerbations; therefore, tobramycin desensitization was not considered a sufficiently effective rescue strategy for this acute episode. Polymyxin B was avoided because of prior immediate throat numbness and dyspnea, and inhaled colistin was not pursued because of concern for bronchospasm or respiratory intolerance in the setting of severe respiratory failure. Given these limitations, the proposed desensitization protocol underwent multidisciplinary review involving respiratory specialists and clinical pharmacists. After assessment of the potential risks, benefits, alternative therapeutic options, and monitoring requirements, the multidisciplinary team determined that rapid intravenous desensitization to CAZ‐AVI (2.5 g/100 mL; ceftazidime 2 g and avibactam 0.5 g) represented the most appropriate therapeutic option. Detailed discussion was conducted with the patient and her family, and informed consent was obtained before treatment initiation.

Desensitization was performed in a closely monitored setting with continuous surveillance of vital signs. Emergency medications, including epinephrine and antihistamines, as well as airway management equipment, were readily available. Given the patient's reproducible urticarial reactions that had previously responded promptly to desloratadine, desloratadine 5 mg was administered 2 h before desensitization after individualized risk–benefit assessment; this was not considered a routine component of the protocol. A stepwise dose‐escalation protocol was implemented, beginning with an extremely low initial dose and gradually increasing to the full therapeutic dose (see Table 1). During the procedure, the patient experienced only mild transient pruritus, which resolved spontaneously without intervention. No severe adverse reactions occurred.

**TABLE 1:** CAZ‐AVI 13‐step desensitization protocol.

Step	Solution bag	Concentration (mg/mL)	Infusion rate (mL/h)	Time (min)	Volume per step (mL)	Dose per step (mg)	Cumulative dose (mg)
1	Bag 1 (1:50)	0.50	2.00	15	0.50	0.25	0.25
2	Bag 1 (1:50)	0.50	5.00	15	1.25	0.63	0.88
3	Bag 1 (1:50)	0.50	10.00	15	2.50	1.25	2.13
4	Bag 1 (1:50)	0.50	20.00	15	5.00	2.50	4.63
5	Bag 2 (1:5)	5.00	5.00	15	1.25	6.25	10.88
6	Bag 2 (1:5)	5.00	10.00	15	2.50	12.50	23.38
7	Bag 2 (1:5)	5.00	20.00	15	5.00	25.00	48.38
8	Bag 2 (1:5)	5.00	40.00	15	10.00	50.00	98.38
9	Bag 3	25.00	10.00	15	2.50	62.50	160.88
10	Bag 3	25.00	20.00	15	5.00	125.00	285.88
11	Bag 3	25.00	40.00	15	10.00	250.00	535.88
12	Bag 3	25.00	80.00	15	20.00	500.00	1035.88
13^a^	Bag 3	25.00	80.00	44	60.50	1464.00	2500.00

^a^ Bag 3 was the prefilled infusion bag; steps 1–12 represent dose‐escalation steps administered at 15‐min intervals; step 13 represents the final full‐dose infusion with Bag 3 to complete the cumulative target dose of 2.5 g.

## 4 Conclusions and Results (Outcome and Follow‐Up)

Following completion of desensitization, the patient successfully received a 14‐day course of CAZ‐AVI at the standard therapeutic dose. Her body temperature normalized, purulent sputum production decreased, dyspnea gradually improved, and hemoptysis did not recur. Her overall condition improved clinically, and inflammatory markers declined significantly (Table 2). The patient was discharged in stable condition after 17 days of hospitalization. During a total follow‐up of 7 weeks after the index hospitalization, no recurrent hypersensitivity reactions were observed. Approximately 2 weeks after discharge, the patient required a second course of CAZ‐AVI for recurrent pulmonary infection. Because rapid desensitization induces temporary rather than permanent tolerance, the same desensitization protocol was repeated before re‐exposure. She completed the additional 2‐week CAZ‐AVI course without severe hypersensitivity reactions and remained clinically stable for a further 3 weeks.

**TABLE 2:** Clinical and laboratory parameters before and after CAZ‐AVI therapy.

Parameter	Before treatment	After treatment
PaCO_2_ (mmHg)^a^	60.8	39
PaO_2_ (mmHg)^a^	46.9	83.7
SpO_2_ (%)^a^	82	94
Oxygenation index	223.33	398.57
White blood cell count (×10^9^/L)	16.84	8.61
Neutrophil count (×10^9^/L)	14.5	3.73
Neutrophil percentage (%)	86.1	43.3
C‐reactive protein (mg/L)	146.45	2.73

^a^ FiO_2_ = 21%.

## 5 Discussion

To our knowledge, only a limited number of cases describing successful desensitization to CAZ‐AVI following immediate hypersensitivity reactions have been reported in the literature [10, 11]. Rapid intravenous desensitization is believed to induce temporary immunologic tolerance through controlled antigen exposure [12]. In this case, the patient's previous reactions to CAZ‐AVI and cefoperazone/sulbactam were clinically suggestive of an immediate hypersensitivity reaction, given the rapid onset of urticaria and flushing shortly after drug administration. The reproducible nature of these reactions and their response to desloratadine further supported this clinical suspicion. Formal allergological evaluation, including skin prick testing, intradermal testing, serum tryptase measurement, specific IgE assays, and basophil activation testing, was not performed because of the patient's critical clinical condition and urgent need for effective antimicrobial therapy. Therefore, although the clinical presentation was suggestive of an immediate hypersensitivity reaction, the exact culprit component could not be definitively determined. The temporal relationship and reproducibility of reactions following CAZ‐AVI and cefoperazone/sulbactam suggested a possible cephalosporin‐related hypersensitivity pattern; however, hypersensitivity to ceftazidime, the avibactam component, or formulation excipients cannot be distinguished in this case. Furthermore, the patient's reaction to polymyxin B raises the possibility of multiple drug sensitizations or alternative non‐IgE‐mediated mechanisms, including mast‐cell activation, infusion‐related reactions, complement‐mediated reactions, or reactions to excipients. The underlying immunologic mechanism therefore remains uncertain.

During the desensitization procedure, a brief episode of mild pruritus occurred and resolved spontaneously, without progression to systemic symptoms. This case underscores the importance of structured rapid desensitization protocols in critically ill CF patients when first‐line therapy is limited by drug hypersensitivity. In individuals with MDR *P. aeruginosa* pneumonia and multiple antimicrobial allergies, desensitization may allow continuation of the preferred therapy despite the theoretical risk of severe allergic reactions. It is important to emphasize that desensitization induces temporary tolerance only; interruption of therapy for more than 24 h may require repeat desensitization [8].

Antihistamine premedication is generally not recommended in standard rapid drug desensitization (RDD) protocols because it may suppress early cutaneous manifestations and delay recognition of evolving systemic hypersensitivity or anaphylaxis [8]. Nevertheless, individualized protocol modifications, including selective premedication, have been described in high‐risk clinical settings after careful risk–benefit assessment [8, 13, 14]. In the present case, desloratadine 5 mg was administered 2 h before desensitization as an individualized decision, based on the patient's reproducible urticarial reactions that had previously responded promptly to desloratadine, her severe respiratory failure, and the lack of equally effective antimicrobial alternatives. We acknowledge that this approach may reduce the sensitivity of early cutaneous warning signs; therefore, desensitization was performed under continuous cardiopulmonary monitoring with immediate availability of emergency medications, airway management equipment, and experienced clinical staff. The absence of urticaria or flushing during escalation was not interpreted as absence of systemic hypersensitivity progression, and this approach should not be regarded as a general recommendation for routine premedication during RDD.

In patients with CF, impaired mucociliary clearance leads to retention of thick airway secretions, chronic bacterial colonization, and recurrent pulmonary exacerbations [2]. Chronic mucoid *P. aeruginosa* infection often requires repeated intravenous antibiotic courses, and frequent antibiotic exposure is a recognized risk factor for drug hypersensitivity [15]. Previous studies have reported that up to 20% of adults with CF develop hypersensitivity reactions to β‐lactam antibiotics [16]. In the present case, the patient's extensive history of intravenous antibiotic therapy likely contributed to her clinically suspected immediate hypersensitivity to CAZ‐AVI. Although CFTR modulator therapy has become standard of care in many countries and may reduce pulmonary exacerbations and chronic *P. aeruginosa* infection burden in eligible patients [17, 18]. Ivacaftor and elexacaftor/tezacaftor/ivacaftor combinations were not formally approved or routinely accessible in mainland China during the treatment period, including at our institution. The patient carried a homozygous p.R334W residual‐function variant that may theoretically demonstrate responsiveness to ivacaftor‐based therapy; however, genotype‐guided CFTR modulation could not be implemented because of regional accessibility limitations. Therefore, in this acute episode of MDR *P. aeruginosa* pneumonia with limited therapeutic alternatives, rapid desensitization was considered necessary to allow administration of the preferred active antimicrobial agent.

Pharmacokinetic considerations are important in CF because hydrophilic antimicrobials, including β‐lactams, may show altered distribution and clearance in this population [19]. The standard CAZ‐AVI regimen of 2.5 g every 8 h was selected because the patient had preserved renal function, the regimen is approved for adults with normal renal function, and PK/PD data in adult CF pulmonary exacerbations support this dosing strategy [20]. Therapeutic drug monitoring was considered but was not performed because CAZ‐AVI concentration monitoring was not routinely available at our institution.

In this report, a 13‐step protocol was implemented, consisting of 12 dose‐escalation steps administered at 15‐min intervals followed by one final full‐dose infusion step to complete the cumulative target dose of 2.5 g. The 15‐min interval was based on the widely used Castells rapid desensitization framework, which has subsequently been adapted for β‐lactam antibiotics and other antimicrobial agents [13, 21]. According to the product information for CAZ‐AVI (Zavicefta), the recommended therapeutic concentration range is 8–40 mg/mL, with 25 mg/mL commonly used in clinical practice. In our protocol, the highest concentration corresponded to 25 mg/mL, remaining within the recommended therapeutic range. In contrast, the protocol described by Coop and Berg [10] reached concentrations of up to 50 mg/mL and achieved a shorter total infusion time of 187 min, exceeding the usual therapeutic concentration range. Our total infusion time was 252 min, after which treatment was continued at the final concentration of 25 mg/mL, similar to the protocol reported by Cristallo et al. [11] with successful desensitization. Maintaining the infusion within the recommended concentration range may provide an additional safety margin. An additional practical advantage of this protocol is its compatibility with commercially available prefilled ready‐to‐use infusion bags (2.5 g/100 mL), as used in our case, as well as reconstituted vial formulations. Because the final therapeutic concentration corresponded to the undiluted commercial preparation, only two diluted solutions were required: a 1:5 dilution (prepared by diluting the original solution 1:4 with normal saline) and a 1:50 dilution (prepared by diluting the 1:5 solution 1:9 with normal saline). Compared with previously published protocols [11] requiring preparation of multiple dilution levels, this approach simplifies solution preparation and may facilitate broader clinical implementation.

After clinical recovery, referral for comprehensive allergological evaluation should be considered, particularly if future re‐exposure to CAZ‐AVI or related agents may become necessary. Such evaluation may include skin prick testing, intradermal testing, serum tryptase measurement, specific IgE assays, and basophil activation testing where available. In regions where specialized allergy and immunology services are limited, structured rapid desensitization protocols may nevertheless provide a pragmatic therapeutic option when urgently needed. The patient was advised to undergo formal allergy evaluation in the future. The repeated desensitization before subsequent CAZ‐AVI re‐exposure further supports that rapid drug desensitization induces temporary rather than permanent tolerance.

## 6 Conclusion

In patients with CF complicated by MDR *P. aeruginosa* infection and clinically suspected immediate hypersensitivity to CAZ‐AVI, rapid desensitization may represent a safe and effective rescue strategy when conducted under close monitoring and multidisciplinary supervision.

## Author Contributions

**Zuoren Zhou:** conceptualization, data curation, investigation, methodology, visualization, writing – original draft. **Jinqi Zhu:** conceptualization, project administration, supervision, writing – review and editing. **Ge Wu:** methodology, writing – review and editing. **Xi Kang:** investigation, writing – review and editing. **Qing Wang:** investigation, writing – review and editing. **Jie Liu:** investigation, writing – review and editing. **Yan Jiang:** investigation, writing – review and editing.

## Funding

The authors have nothing to report.

## Consent

Written informed consent was obtained from the patient for publication of this case report.

## Conflicts of Interest

The authors declare no conflicts of interest.

## Data Availability

The authors have nothing to report.

## References

[1] J. S. Elborn , “Cystic Fibrosis,” Lancet 388, no. 10059 (2016): 2519–2531. 27140670 10.1016/S0140-6736(16)00576-6

[2] E. Rossi , R. La Rosa , J. A. Bartell , et al., “ *Pseudomonas aeruginosa* Adaptation and Evolution in Patients With Cystic Fibrosis,” Nature Reviews Microbiology 19, no. 5 (2021): 331–342. 33214718 10.1038/s41579-020-00477-5

[3] O. Ciofu and T. Tolker‐Nielsen , “Tolerance and Resistance of *Pseudomonas aeruginosa* Biofilms to Antimicrobial Agents‐How *P. aeruginosa* Can Escape Antibiotics,” Frontiers in Microbiology 10 (2019): 913. 31130925 10.3389/fmicb.2019.00913 PMC6509751

[4] J. Reyes , L. Komarow , L. Chen , et al., “Global Epidemiology and Clinical Outcomes of Carbapenem‐Resistant *Pseudomonas aeruginosa* and Associated Carbapenemases (POP): A Prospective Cohort Study,” Lancet Microbe 4, no. 3 (2023): e159–e170. 36774938 10.1016/S2666-5247(22)00329-9 PMC10016089

[5] Y. Zhang , X. L. Chen , A. W. Huang , et al., “Mortality Attributable to Carbapenem‐Resistant *Pseudomonas aeruginosa* Bacteremia: A Meta‐Analysis of Cohort Studies,” Emerging Microbes & Infections 5, no. 3 (2016): e27. 27004762 10.1038/emi.2016.22 PMC4820673

[6] M. Shirley , “Ceftazidime‐Avibactam: A Review in the Treatment of Serious Gram‐Negative Bacterial Infections,” Drugs 78, no. 6 (2018): 675–692. 29671219 10.1007/s40265-018-0902-x

[7] J. P. Horcajada , M. Montero , A. Oliver , et al., “Epidemiology and Treatment of Multidrug‐Resistant and Extensively Drug‐Resistant *Pseudomonas aeruginosa* Infections,” Clinical Microbiology Reviews 32, no. 4 (2019): e00031‐19. 31462403 10.1128/CMR.00031-19 PMC6730496

[8] E. Alvarez‐Cuesta , R. Madrigal‐Burgaleta , A. D. Broyles , et al., “Standards for Practical Intravenous Rapid Drug Desensitization & Delabeling: A WAO Committee Statement,” World Allergy Organization Journal 15, no. 6 (2022): 100640. 35694005 10.1016/j.waojou.2022.100640 PMC9163606

[9] B. Yang , C. Lei , D. Yang , Z. Tan , T. Guo , and H. Luo , “Whole‐Exome Sequencing Identified CFTR Variants in Two Consanguineous Families in China,” Frontiers in Genetics 12 (2021): 631221. 34276759 10.3389/fgene.2021.631221 PMC8283821

[10] C. C. A. Coop and M. J. R. Berg , “Rapid Desensitization After a Type I Hypersensitivity Reaction to Ceftazidime/Avibactam,” Federal Practitioner 39, no. 2 (2022): 94–96. 35444389 10.12788/fp.0220 PMC9014940

[11] M. Cristallo , N. Chaoul , M. Albanesi , E. Nettis , and A. Di Girolamo , “Allergy to CAZ‐AVI: Diagnosis and Desensitization,” Postepy Dermatologii I Alergologii 41, no. 2 (2024): 239–241. 38784929 10.5114/ada.2024.138836 PMC11110221

[12] H. J. Legere, 3rd , R. I. Palis , T. Rodriguez Bouza , A. Z. Uluer , and M. C. Castells , “A Safe Protocol for Rapid Desensitization in Patients With Cystic Fibrosis and Antibiotic Hypersensitivity,” Journal of Cystic Fibrosis 8, no. 6 (2009): 418–424. 19740711 10.1016/j.jcf.2009.08.002 PMC2787787

[13] A. D. Broyles , A. Banerji , S. Barmettler , et al., “Practical Guidance for the Evaluation and Management of Drug Hypersensitivity: Specific Drugs,” Journal of Allergy and Clinical Immunology: In Practice 8, no. 9S (2020): S16–S116. 33039007 10.1016/j.jaip.2020.08.006

[14] P. Lopez‐Gonzalez , R. Madrigal‐Burgaleta , L. V. Carpio‐Escalona , et al., “Assessment of Antihistamines and Corticosteroids as Premedication in Rapid Drug Desensitization to Paclitaxel: Outcomes in 155 Procedures,” Journal of Allergy and Clinical Immunology. In Practice 6, no. 4 (2018): 1356–1362. 29248386 10.1016/j.jaip.2017.11.013

[15] B. Y. Thong and T. C. Tan , “Epidemiology and Risk Factors for Drug Allergy,” British Journal of Clinical Pharmacology 71, no. 5 (2011): 684–700. 21480948 10.1111/j.1365-2125.2010.03774.x PMC3093074

[16] J. A. Burrows , L. M. Nissen , C. M. Kirkpatrick , and S. C. Bell , “β‐Lactam Allergy in Adults With Cystic Fibrosis,” Journal of Cystic Fibrosis 6, no. 4 (2007): 297–303. 17182289 10.1016/j.jcf.2006.11.001

[17] H. G. M. Heijerman , E. F. McKone , D. G. Downey , et al., “Efficacy and Safety of the Elexacaftor Plus Tezacaftor Plus Ivacaftor Combination Regimen in People With Cystic Fibrosis Homozygous for the F508del Mutation: A Double‐Blind, Randomised, Phase 3 Trial,” Lancet 394, no. 10212 (2019): 1940–1948. 31679946 10.1016/S0140-6736(19)32597-8 PMC7571408

[18] P. G. Middleton , M. A. Mall , P. Drevinek , et al., “Elexacaftor‐Tezacaftor‐Ivacaftor for Cystic Fibrosis With a Single Phe508del Allele,” New England Journal of Medicine 381, no. 19 (2019): 1809–1819. 31697873 10.1056/NEJMoa1908639 PMC7282384

[19] A. M. Akkerman‐Nijland , O. W. Akkerman , F. Grasmeijer , et al., “The Pharmacokinetics of Antibiotics in Cystic Fibrosis,” Expert Opinion on Drug Metabolism & Toxicology 17, no. 1 (2021): 53–68. 33213220 10.1080/17425255.2021.1836157

[20] T. J. Bensman , J. Wang , J. Jayne , et al., “Pharmacokinetic‐Pharmacodynamic Target Attainment Analyses to Determine Optimal Dosing of Ceftazidime‐Avibactam for the Treatment of Acute Pulmonary Exacerbations in Patients With Cystic Fibrosis,” Antimicrobial Agents and Chemotherapy 61, no. 10 (2017): e00988‐17. 28784670 10.1128/AAC.00988-17 PMC5610479

[21] M. Castells , “Rapid Desensitization for Hypersensitivity Reactions to Medications,” Immunology and Allergy Clinics of North America 29, no. 3 (2009): 585–606. 19563999 10.1016/j.iac.2009.04.012

